# The relationship between literacy and multimorbidity in a primary care setting

**DOI:** 10.1186/1471-2296-13-33

**Published:** 2012-07-03

**Authors:** Catherine Hudon, Martin Fortin, Marie-Eve Poitras, José Almirall

**Affiliations:** 1 Département de médecine de famille, Université de Sherbrooke, Québec, Canada; 2 Centre de santé et de services sociaux de Chicoutimi, Québec, Canada

## Abstract

**Background:**

Multimorbidity is now acknowledged as a research priority in primary care. The identification of risk factors and people most at risk is an important step in guiding prevention and intervention strategies. The aim of this study was to examine the relationship between literacy and multimorbidity while controlling for potential confounders.

**Methods:**

Participants were adult patients attending the family medicine clinic of a regional health centre in Saguenay (Quebec), Canada. Literacy was measured with the Newest Vital Sign (NVS). Multimorbidity was measured with the Disease Burden Morbidity Assessment (DBMA) by self-report. Information on potential confounders (age, sex, education and family income) was also collected. The association between literacy (independent variable) and multimorbidity was examined in bivariate and multivariate analyses. Two operational definitions of multimorbidity were used successively as the dependent variable; confounding variables were introduced into the model as potential predictors.

**Results:**

One hundred three patients (36 men) 19–83 years old were recruited; 41.8% had completed 12 years of school or less. Forty-seven percent of patients provided fewer than four correct answers on the NVS (possible low literacy) whereas 53% had four correct responses or more. Literacy and multimorbidity were associated in bivariate analyses (p < 0.01) but not in multivariate analyses, including age and family income.

**Conclusion:**

This study suggests that there is no relationship between literacy and multimorbidity when controlling for age and family income.

## Background

Multimorbidity, the coexistence of two or more chronic diseases in the same patient, has received growing interest in the primary care literature over the past few years and is now acknowledged by many as a research priority [1-6]. Patients with multimorbidity seen in family practice represent the rule rather than the exception [7-9]. These patients are more likely to present poor health outcomes such as a decrease in quality of life [10], increased psychological distress [11], more postsurgical complications [12], longer hospitalisations [13,14], and higher rates of mortality [15,16].

In the wake of prevention and intervention strategies to address this issue, the identification of risk factors and of people most at risk is an important aspect. Aging [7,9] and low socioeconomic status [8,9,17,18] are already clearly associated with an increased incidence of multimorbidity. Without directly measuring multimorbidity, many studies have also assessed a relationship between low literacy and poorer health status using global health measures [19-26] which raises the possibility of an independent association between literacy or health literacy and multimorbidity.

The Institute of Medicine defines health literacy as “the degree to which individuals have the capacity to obtain, process and understand basic health information and services needed to make appropriate health decisions” [27]. This definition presents health literacy as a set of individual capacities that allow the person to acquire and use new information [28]. Others have argued that health literacy is the ability to function in the health care environment and depends on characteristics of both the individual and the health care system [28]. In both cases, individual capacity, including reading fluency and prior knowledge, have an influence on health literacy [28]. Reading fluency, or literacy [29], is the ability to mentally process written materials and form new knowledge. The first National Adult Literacy Study [30] divided literacy into three skill sets: 1) the ability to read and understand text; 2) the ability to locate and use information in documents; and 3) the ability to apply arithmetic operations and use numerical information in printed materials.

Notwithstanding the interest in looking into a potential association between health literacy and different health outcomes such as multimorbidity, health literacy is easier to conceptualize than to measure. Even if valid measures of literacy exist, a measure that accurately depicts the multiple dimensions of health literacy is still needed [28,31]. Most studies measuring an association with outcomes used a measure of literacy.

A low level of literacy has been linked to several negative health outcomes in patients: poorer knowledge of their health problems [32-35], poorer health status, and higher hospitalization rates [36,37], than those having an adequate level of literacy. Inadequate literacy has also been associated with problems with the use of preventive services [38], delayed diagnoses [39], and all-cause mortality among community dwelling older aged people [40,41].

The aim of this study was to evaluate the relationship between literacy and multimorbidity while controlling for potential confounders.

## Methods

### Study design and setting

A descriptive study was conducted among patients attending a family medicine clinic of a regional health centre in Saguenay (Québec), Canada. The Saguenay region’s population is approximately 150,000 living in one major city and several smaller boroughs. The Saguenay region has about 130 general practitioners, nearly 80% of whom have a general practice and work in a private office or a public institution. The present study was carried out in an institutional practice (Centre de santé et de services sociaux de Chicoutimi) where 12 family doctors and four nurses provide their services to 9,000 registered patients.

### Participants and sampling

The sampling process took place during 10 four-hour sessions over a two-week period. Patients solicited were asked to complete a short questionnaire to determine eligibility. To be included in the study, participants had to be aged 18 years or older and regular patients of the family medicine clinic.

Patients were excluded if they presented an unstable acute or psychiatric condition, or if they were pregnant or unable to provide informed consent. Non eligible patients were thanked for completing the short questionnaire for eligibility and no further action was taken with them.

### Instruments

Literacy was measured with the Newest Vital Sign (NVS) [42]. The NVS consists of a nutrition label from an ice cream container. The label is given to the patient, who is then asked six questions about it. The first four questions require document and quantitative skills, including the ability to calculate percentages. It takes approximately three minutes to administer. The NVS is reliable (Cronbach alpha > 0.76) and correlated with the Test of Functional Health Literacy in Adults (TOFHLA), another instrument measuring literacy [42]. The NVS is a markedly better predictor of patients with low literacy than education or age alone. Patients with four correct responses or more are considered as having adequate literacy whereas fewer than four correct answers indicate low literacy. The NVS was treated as a dichotomous variable: 1) low literacy (NVS < 4), and 2) adequate literacy (NVS ≥ 4) [42].

Multimorbidity was measured with a simplified version of the Disease Burden Morbidity Assessment (DBMA) by self-report described by Bayliss and colleagues [43]. The original instrument includes 21 diseases. For this study, only 11 diseases with a high prevalence in our setting were kept in order to reduce the time needed to complete the questionnaire: hypertension, hyperlipidemia, asthma, chronic obstructive pulmonary disease, diabetes, osteoarthritis, back pain, other musculoskeletal conditions, overweight, angina/coronary artery disease, and congestive heart failure. To assess their disease burden, patients identified in the list of 11 conditions those that they had, and rated the interference of each condition with daily activities on a five-point scale from 1 (“not at all”) to 5 (“a lot”). The total multimorbidity score is the sum of conditions weighed by the level of interference assigned to each. The original DBMA was found to be strongly associated with subjective health status [43].

Two operational definitions of multimorbidity were used: 1) the DBMA score while considering all 11 conditions (DBMA 11); 2) the DBMA score while taking into account only six conditions, which were considered to be associated with lifestyle habits or that have been reported as independently associated with inadequate literacy [44,45]: hypertension, hyperlipidemia, diabetes mellitus, overweight, angina/coronary artery disease, and congestive heart failure (DBMA 6). The latter operational definition of multimorbidity was computed to have a multimorbidity measure that, at least theoretically, could be associated with literacy.

### Data collection

The patients were recruited in the waiting room of a family medicine clinic. A research assistant approached each patient to explain the project. All subjects who refused to participate did it at this point, and no data were collected from them. Patients who agreed to participate and met all eligibility criteria were brought to a small separate room where the research assistant obtained their written consent, collected sociodemographic data (sex, age, education and family income), and administered both the NVS and the DBMA. The responses to the NVS were collected by the research assistant while the DBMA questionnaire was completed by the patient in the presence of the research assistant who helped them, if needed (for example, reading the questionnaire to patients who did not have their glasses). Filling out the questionnaires took no more than 10 min. Participants did not receive any payment for participating in the study.

The study was approved by the research ethics board of the Centre de santé et de services sociaux de Chicoutimi.

### Data analysis

The associations between multimorbidity (dependent variable) and the other variables (age, sex, education, family income and NVS) were first examined in bivariate analyses. In a multivariate general linear modeling analysis, both operational definitions of multimorbidity were used successively as dependent variable, and the variables with a significant relationship with multimorbidity in the bivariate analysis were used as potential predictors in the model. Correlations between predictor variables were examined to determine the presence of collinearity.

All analyses were performed with PASW Statistics 18 (SPSS Inc.). The α significance level was set at 0.05.

## Results

A total of 103 patients (36 men) between 19 and 83 years of age participated in the study; 41.8% had a 12-year or less school education and 30.1% had completed university studies (Table 1). Forty-eight percent of patients had a NVS score within the range of limited literacy (NVS < 4), and 52% had adequate health literacy. The number of subjects with and without multimorbidity was well distributed across the sample (Table 1).

**Table 1:** Characteristics of the sample

**Characteristic**	**Participants n = 103**
Mean (SD) age, years	49.9 (7.1)
Male, %	35.0
NVS < 4, %	47.6
NVS ≥ 4, %	52.4
DBMA 11. Mean (SD)	5.8 (6.5)
DBMA 6, Mean (SD)	2.5 (3.2)
0 or 1 disease, %	42.7
2 or more diseases, %	57.3
Education, %	
≤ 12 years	41.8
College	27.2
University	30.1
Missing data	1.0
Household income in Canadian dollars, %	
< $10,000	5.8
$10,000-$29,999	18.4
$30,000-$49,999	31.0
≥ $50,000	41.7
Missing data	2.9

**Figure 1:**
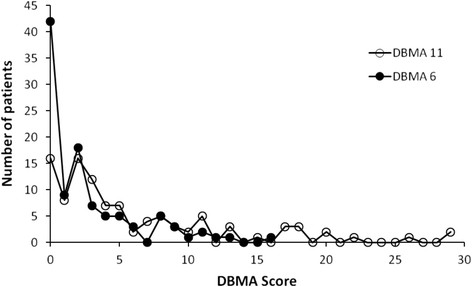
**Distribution of scores on operational definitions of multimorbidity.** DBMA = Disease Burden Morbidity Assessment (see text for description of chronic conditions considered in DBMA 11 and DBMA 6).

The scores of both operational definitions of multimorbidity showed a wide distribution across the sample. Figure 1 shows the distribution of each definition of multimorbidity score. As expected, the DBMA 11 had the widest variation, with a range of 0–29 and a median of three; the DBMA 6 varied from 0–13 with a median of two. The distribution of both operational definitions of multimorbidity followed a similar trend, the most striking difference being that 16 patients (15.5%) had a DBMA 11 score of zero whereas the number of patients with a DBMA 6 score of zero was 42 (40.8). This was also an expected finding due to the smaller number of chronic conditions considered in the DBMA 6.

In bivariate analyses, age, family income, and literacy were linked to both operational definitions of multimorbidity. There was no association between sex or education and multimorbidity (Table 2).

**Table 2:** Results of bivariate analyses

	**Multimorbidity measure**			
**Variable**	**DBMA 11**		**DBMA 6**	
	**β†**	***p* value**	**β†**	***p* value**
Age	0.439	< 0.01	0.490	< 0.01
Sex	−0.004	0.97	0.061	0.55
Education	−0.185	0.06	−0.039	0.70
Income	−0.394	< 0.01	−0.286	< 0.01
NVS	−0.334	< 0.01	−0.214	0.04

**†** β **=** regression coefficient.

In multivariate analyses, only age and family income were associated with both operational definitions of multimorbidity (Table 3). Literacy was no longer related to multimorbidity when age and family income were introduced into the model. No collinearity was found among the predictor variables.

**Table 3:** Results of multivariate analyses

	**Multimorbidity measure**			
	**DBMA 11**		**DBMA 6**	
**Variable**	**β†**	**p value**	**β†**	**p value**
Age	0.40	< 0.01	0.47	< 0.01
Sex	−0.02	0.82	0.02	0.83
Education	−0.07	0.46	0.03	0.76
Income	−0.31	< 0.01	−0.21	0.02
NVS	−0.08	0.41	−0.02	0.87

**†** β **=** regression coefficient.

## Discussion

To our knowledge, this is the first publication addressing the relationship between literacy and multimorbidity. The results of the study suggest that low literacy is associated with the presence of multimorbidity in adults consulting in primary care in bivariate analysis, but this association is no longer present when controlling for age and family income.

Many previous studies have indicated that patients with low literacy were more likely to report poorer health than patients with adequate literacy [19-26], while other studies found no relationship between literacy and health status when controlling for education [46,47]. Many studies were conducted in older aged patients [21,23-25,46,47] making generalization of results to other age groups difficult. One study did not adjust for confounding variables [21]. In another study [20], literacy was not evaluated with a validated tool but by the staff of the institution where the study was carried out. In all studies, health status was evaluated globally using self-rated health status categories. In this research, we used a more detailed instrument to measure patients’ disease burden than the studies supporting the existence of a relationship between health literacy and global health measures.

Other research has addressed the relationship between literacy and specific diseases with inconsistent results. One study reported that inadequate literacy (measured with the short form of the Test of Functional Health Literacy in Adults) was an independent predictor of diabetes mellitus and heart failure but not hypertension, arthritis or pulmonary disease, while adjusting for sociodemographic variables (age, sex, race/ethnicity, education and annual income), health risk behaviours (smoking habit and alcohol use), and body mass index [45]. Another study using the same measure of literacy found that heart attack, stroke, hypertension, diabetes, arthritis and depression were all associated with the literacy level [48]. A study accounting for hypertension, diabetes, obesity and depression concluded that only depression remained significantly associated with literacy after adjusting for confounders [46]. Another study reported that individuals with low literacy had significantly higher rates of arthritis and hypertension, but no statistical differences were found in the prevalence of diabetes, pulmonary, or heart disease [25].

A low level of literacy may be linked to certain health issues and not others. As our measure of multimorbidity evaluated chronic diseases as a whole as well as their severity, we may not have detected a link because of the presence of specific health issues not associated with literacy. An association between literacy and multimorbidity may exist when two or more specific diseases individually related to health literacy coexist in one person. The conceptualization and measure of multimorbidity could therefore have an impact on this association. That is why we conducted analysis using two distinctive conceptualizations and measures, in order to verify if results were different. It was not the case.

We found that multimorbidity was associated with age and family income in the multivariate models. The association of multimorbidity with age is well recognized [7-9,49]. The relationship between socioeconomic status and multimorbidity has also been extensively documented [8,9,17,18].

Our results do not allow us to rule on a potential association between health literacy, a more global concept than literacy [28,31], and multimorbidity. A comprehensive measure of health literacy that considers other dimensions of the concept still needs to be developed. We could then verify if there is a link between health literacy and multimorbidity. Although we did not observe a direct association between literacy and multimorbidity, it is still important to continue taking this variable into account in patient care in order to tailor health information to patient needs and in a format they can understand [42].

A limitation of this study is that participants were not randomly selected from the general population. We recruited patients from the waiting room of a single primary care setting. This method may over sample complex patients with several diseases or frequent attendees. However, we were able to recruit a group of patients with a good distribution of multimorbidity and literacy. Another limitation is the lack of statistical power to carry out multivariate analysis by individual disease. Although the study was conducted in one family practice, we expect the same results from similar primary care settings.

In conclusion, this study suggests that there is no relationship between literacy and multimorbidity when controlling for age and family income. Patients with multimorbidity may have specific diseases that are associated with low literacy. Further studies are needed to identify individual diseases and combinations of diseases linked to literacy while controlling for potential confounding variables. A possible association between health literacy and multimorbidity still needs to be explored when a comprehensive measure of health literacy is available.

## Competing interests

The authors declare that they have no competing interests.

## Authors’ contributions

CH, MF and M-EP conceived and designed the study. MF supervised the data collection, participated in the data analysis and helped draft the manuscript. M-EP participated in the data collection and analysis and critically reviewed the manuscript. JA and CH analyzed the data and drafted the manuscript. All authors read and gave their final approval of the version of the manuscript submitted for publication. CH takes responsibility for the integrity of the work as a whole.

## Pre-publication history

The pre-publication history for this paper can be accessed here:

http://www.biomedcentral.com/1471-2296/13/33/prepub
